# Clinical Application of 3D-Assisted Surgery Techniques in Treatment of Intra-Articular Distal Radius Fractures: A Systematic Review in 718 Patients

**DOI:** 10.3390/jcm13237296

**Published:** 2024-11-30

**Authors:** Lisanne J. M. Roelofs, Nick Assink, Joep Kraeima, Kaj ten Duis, Job N. Doornberg, Jean-Paul P. M. de Vries, Anne M. L. Meesters, Frank F. A. IJpma

**Affiliations:** 1 Department of Surgery, Division of Trauma Surgery, University of Groningen, University Medical Center Groningen, 9700 VB Groningen, The Netherlands; l.j.m.roelofs@umcg.nl (L.J.M.R.); n.assink@umcg.nl (N.A.); k.ten.duis@umcg.nl (K.t.D.); a.m.l.meesters@umcg.nl (A.M.L.M.); 2 3D Lab and Department of Oral and Maxillofacial Surgery, University of Groningen, University Medical Center Groningen, 9700 VB Groningen, The Netherlands; j.kraeima@umcg.nl; 3 Department of Orthopaedic Surgery, University of Groningen, University Medical Center Groningen, 9700 VB Groningen, The Netherlands; j.n.doornberg@umcg.nl; 4 Department of Orthopaedic Trauma, Flinders Medical Centre, GPO Box 2100, Adelaide, SA 5042, Australia; 5 Department of Surgery, Division of Vascular Surgery, University of Groningen, University Medical Center Groningen, 9700 VB Groningen, The Netherlands; j.p.p.m.de.vries@umcg.nl

**Keywords:** distal radius, fracture, 3D, three-dimensional, 3D-assisted, 3D virtual surgical planning, 3D printing

## Abstract

**Objectives**: Three-dimensional (3D) technology is increasingly applied in the surgical treatment of distal radial fractures and may optimize surgical planning, improve fracture reduction, facilitate implant and screw positioning, and thus prevent surgical complications. The main research questions of this review were as follows: (1) “How do 3D-assisted versus 2D-assisted distal radius fracture surgery compare in terms of intraoperative metrics (i.e., operation time and fluoroscopy frequency)?”, and (2) ”What are the effects of 3D-assisted versus 2D-assisted surgery on postoperative outcomes (patient-reported outcome measures (PROMs), range of motion (ROM), fracture reduction, complication rate, and screw placement accuracy)?” **Methods**: This review was performed according to the Preferred Reporting Items for Systematic Reviews (PRISMA) guidelines. In total, 873 articles were found between 1 January 2010 and 1 April 2024, of which 12 (718 patients) were suitable for inclusion. The quality of the studies, assessed using the McMaster quality assessment, ranged from moderate to excellent, although the surgical techniques and outcome measures varied widely. Articles comparing a 3D group to a 2D group (conventional imaging) and reporting on primary or secondary outcomes were included in the analysis, for which weighted means and ranges were calculated. **Results**: Three different concepts of 3D-assisted surgery techniques were identified: (1) 3D virtual surgical planning (VSP), (2) 3D-printed handheld models, and (3) 3D intraoperative guides. Differences between 3D-assisted and conventional 2D-assisted surgery were evaluated. Regarding intraoperative metrics, 3D-assisted surgery significantly reduced operation time by 6 min (weighted mean 66.9 versus 73.2 min) and reduced the fluoroscopy frequency by 1.1 images (5.8 versus 4.7 times). Regarding postoperative outcomes, the weighted mean of the DASH score differed between the 3D- and 2D-assisted groups (17.8 versus 23.9 points), and no differences in PRWE or VAS score were found. Furthermore, our results showed no significant differences in the ROM and fracture reduction parameters. In terms of complications, the application of 3D-assisted surgery decreased the complication rate from 10.7% to 3.6%, and the use of screws with appropriate lengths improved from 75% to 86%. **Conclusions**: Applications of 3D-assisted surgery in distal radial fracture surgery can slightly reduce the operation time and fluoroscopy frequency. Evidence for the improvement of fracture reduction and functional outcomes is still lacking, although it likely reduces the complication rate and improves the use of appropriate screw lengths.

## 1. Introduction

Distal radius fractures are among the most common types of fractures in young adults and the elderly population [1]. A total of 35–85% of patients with these fractures undergo open reduction and internal fixation [2,3,4], mainly depending on their age and address [5]. The aim of surgery is to restore radial alignment, improve joint surface congruency, and provide a stable fixation that allows for early mobilization. A fundamental principle in surgery is “plan your operation, operate your plan”. Conventional radiographs and two-dimensional computed tomography (2D CT) are commonly used for surgical planning. These provide a single-plane view of the fractured bone and lack a three-dimensional (3D) overview, which may make it challenging to plan reduction strategies, including implant and screw positioning. Therefore, 3D technology is increasingly applied in the surgical treatment of distal radial fractures and may optimize surgical planning, improve fracture reduction, facilitate implant and screw positioning, and thereby avoid surgical complications (e.g., screw and/or plate removal due to irritation, secondary fracture displacement, wound infection, carpal tunnel syndrome, wound dehiscence, complex regional pain syndrome, and tendon injury) [6,7]. Understanding and embracing 3D-assisted surgery may be an adjunct for both surgeons and patients due to its potential to enhance precision and outcomes in distal radial fracture surgery [8,9,10,11,12,13,14].

In the last decade, we have witnessed a proliferation of 3D technologies, from 3D-printed models to 3D implants, and from virtual plans to surgical drilling guides [8]. Several types of 3D-assisted surgical techniques are applied in the primary treatment of distal radius fractures. Technologies vary from very simple and low-cost to highly demanding and exclusive. Today, 3D prints are used in many centers to support surgeon–patient communication, resident teaching, and intraoperative visualization, or as a template for the selection and pre-bending of surgical screws and plates [15]. Benefits like a more in-depth understanding of the complex distribution of fracture lines—for example for classification and surgical decision-making—have been described [16,17,18,19]. Clinical application of 3D virtual surgical planning is increasing: a computed tomography (CT) scan of the fracture can be converted into a virtual 3D fracture model for surgical simulation and optimization of the surgical plan [9,20,21,22,23]. Additionally, 3D intraoperative imaging techniques can be used to check and adjust the lengths and positioning of plates and screws before wound closure. Despite the rapid emergence of these innovative 3D techniques, there is a lack of a complete overview and critical appraisal of their use in distal radius fracture treatment.

A systematic review of the existing literature was conducted, with a focus on clinical applications and the added clinical value of 3D-assisted surgical techniques in the treatment of distal radius fractures. First, all identified 3D-assisted surgical techniques were summarized, and secondly, data from the identified studies were analyzed to answer our two research questions: (1) “How do 3D-assisted versus 2D-assisted distal radius fracture surgery compare in terms of intraoperative metrics (i.e., operation time and use of fluoroscopy)?” and (2) “What are the effects of 3D-assisted versus 2D-assisted surgery on postoperative outcomes (patient-reported outcome measures (PROMs), range of motion (ROM), fracture reduction, complication rate and screw placement accuracy)?”.

## 2. Materials and Methods

### 2.1. Protocol

This review was performed according to the Preferred Reporting Items for Systematic Reviews (PRISMA) guidelines [24]. The protocol for this systematic review was registered in the international PROSPERO database (ID 533271). The local Medical Ethical Review Board waived the further need for approval for this study.

### 2.2. Search Strategy

Two databases were searched: Embase and PubMed. Search terms were generated in collaboration with a medical librarian. Table 1 shows the search string, adapted for the search engine of both databases. Articles were included if they were published between 1 January 2010 and 1 March 2024 in order to provide a state-of-the-art overview of 3D-assisted surgery for distal radial fractures.

### 2.3. Study Selection

Study selection was conducted by two observers (LR and FIJ). Articles were imported into Rayyan QCRI [25], which allowed online blinded assessment, and all duplicates were removed. First, all articles were reviewed independently by both observers for inclusion, based on title and abstract. The initial selection was finalized through discussion between both observers and a third observer (NA) until consensus was reached. Studies involving the use of 3D technologies in the surgical management of distal radius fractures were included. Studies of interest focused on surgical treatment of distal radial fractures supported by 3D virtual surgical planning (VSP), 3D-printed handheld fracture models, or the use of 3D intraoperative guides. Study designs deemed suitable for inclusion were randomized controlled trials (RCTs), cohort studies and case series (N > 10). Exclusion criteria were fracture classification studies, cadaver studies, studies with non-human subjects, navigation studies, correction osteotomy studies of malunited (old) fractures, systematic reviews or meta-analyses, case reports, case series (N < 10) and expert opinions. To complete the final study selection, the remaining articles were reviewed and analyzed on a full-text basis by both observers. Differences in assessment were resolved through discussion between the two observers.

### 2.4. Quality Check and Best Evidence Synthesis

The included studies were assessed for methodological quality and risk of bias by following the guidelines of McMaster University Occupational Therapy Evidence-Based Practice Research Group [26]. The articles were independently scored by two authors (LR and AM) and disagreements were resolved by reaching a consensus during a meeting. The McMaster scoring system includes questions regarding the study purpose, literature review, study design, study sample, outcomes, intervention, results, conclusions, and clinical implications. Scores were given with ‘yes’ = 1 point, ‘no’ = 0 points, and not applicable; ‘NA’. The total score reflects the methodological quality with a maximum score of 16 for RCTs, 12 for case series, and 14 for other designs. The definitive score is presented as a percentage that varies from 0 to 100%, with a higher score indicating a higher methodological quality. Studies with a score below 50% were classified as poor, scores between 50–74% as moderate, 75–90% as good and >90% as excellent [26].

Studies that scored 75% or higher on the McMaster quality assessment were considered high-quality in this review, and other studies were considered low-quality. We used best evidence synthesis to ensure that the methodological quality of the original studies was considered. The level of evidence was based on this study’s quality. Evidence levels were considered ‘strong’ when there were consistent findings among multiple high-quality studies, ‘moderate’ when there were consistent findings among multiple low-quality studies and/or one high-quality study, ‘limited’ when based on one low-quality study, ‘conflicting’ when there were inconsistent results among multiple high- and/or low-quality studies, and ‘no evidence’ when none of the above applied [27].

### 2.5. Outcome Measures

First, the methods of applying 3D-assisted surgical techniques for the operative treatment of distal radial fractures were summarized. Then, the data were extracted from the articles. The primary outcomes were intraoperative metrics, defined as (1) operation time (min), and (2) the fluoroscopy frequency (the number of fluoroscopy images taken during the procedure). Secondary outcomes were the postoperative surgical effects, categorized into five groups. First was patient follow-up using PROMs, which include the Disability of the Arm, Shoulder and Hand (DASH) questionnaire [28], the Patient-Rated Wrist Evaluation (PRWE) and pain on the Visual Analogue Scale (VAS) [29]. The second outcome was a measurement of function/range of motion (ROM) (wrist extension, flexion, pronation, supination, and radial and ulnar deviation). These measures can also be combined into the Gartland–Werley score [30,31]. The third outcome was fracture reduction, measured by a gap and/or step-off. A residual intra-articular fracture displacement (gap and/or step-off) of <2 mm was considered clinically acceptable. Additionally, distal radius alignment was measured by palmar tilt, summarizing dorsal tilt (<10 degrees) and volar tilt (<20 degrees)), radial height (<3 mm), radial inclination (>15 degrees) and ulnar variance (<2 mm). The fourth outcome was the postoperative complication rate and the fifth was the accuracy of screw placement, defined as being within 75% of the planned length and position.

### 2.6. Statistical Analysis

Statistical analysis was performed using SPSS software (version 28, IBM, Chicago, IL, USA). Continuous data were presented as means and ranges, whereas rates were presented as percentages. The weighted mean and range were calculated when two or more comparative studies (studies with outcomes of a 2D and a 3D group) reported the outcome variable. Outcome measures from studies that did not compare a 2D and 3D group or were reported in only one article were excluded from these calculations. In all studies, a *p*-value of <0.05 was considered statistically significant.

## 3. Results

### 3.1. Search and Study Characteristics

**Figure 1:**
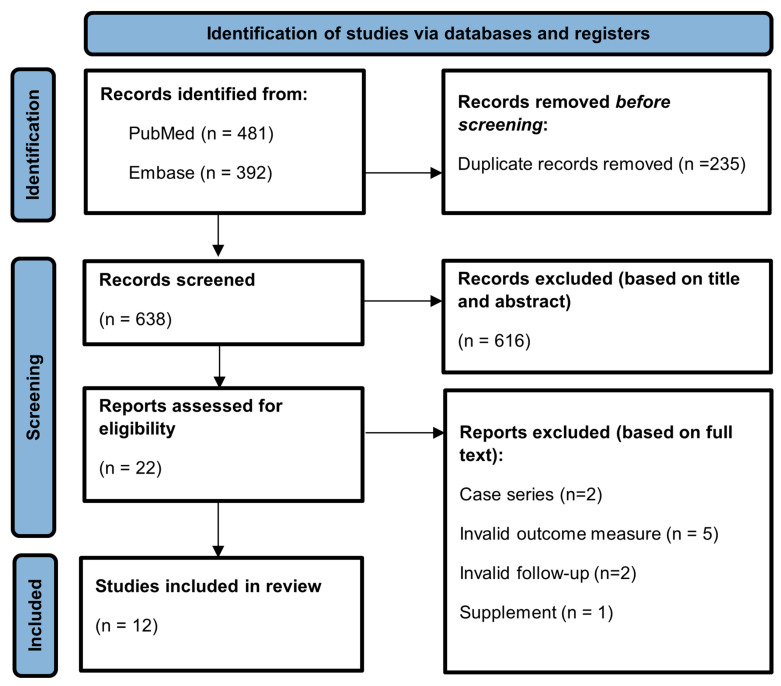
PRISMA flow diagram.

In total, 873 articles were found. After removing duplicates, 638 unique articles were screened based on title and abstract. A total of 22 articles were included for full-text screening. Ten of these full-text articles were excluded for various reasons: case series (n = 2), invalid follow-up (n = 2), invalid outcome measure (n = 5), and supplement to an already included article (n = 1) (Figure 1). Finally, 12 studies met all inclusion criteria and included 718 patients in total (median 48, range 20–171) (Table 2).

### 3.2. Methodological Quality Assessment

Of the twelve included studies, six were randomized controlled trials, two cross-sectional studies, one case–control study and three cohort studies (one retrospective, two prospective). Seven studies compared a 2D- and 3D-surgery group [20,32,33,34,36,37,39], four compared outcomes to the 3D virtual surgical planning [14,38,40,41], and one compared results to the healthy contralateral side [35]. The methodological quality of the studies ranged from moderate to excellent (Table 3), with a median score of 66% (IQR: 56–77%). Five studies were classified as high-quality studies based on the McMaster assessment score [14,20,35,37,38]. These studies are indicated by a ‘✓’ in Table 3.

### 3.3. Identified 3D Applications in Distal Radius Fracture Surgery

**Figure 2:**
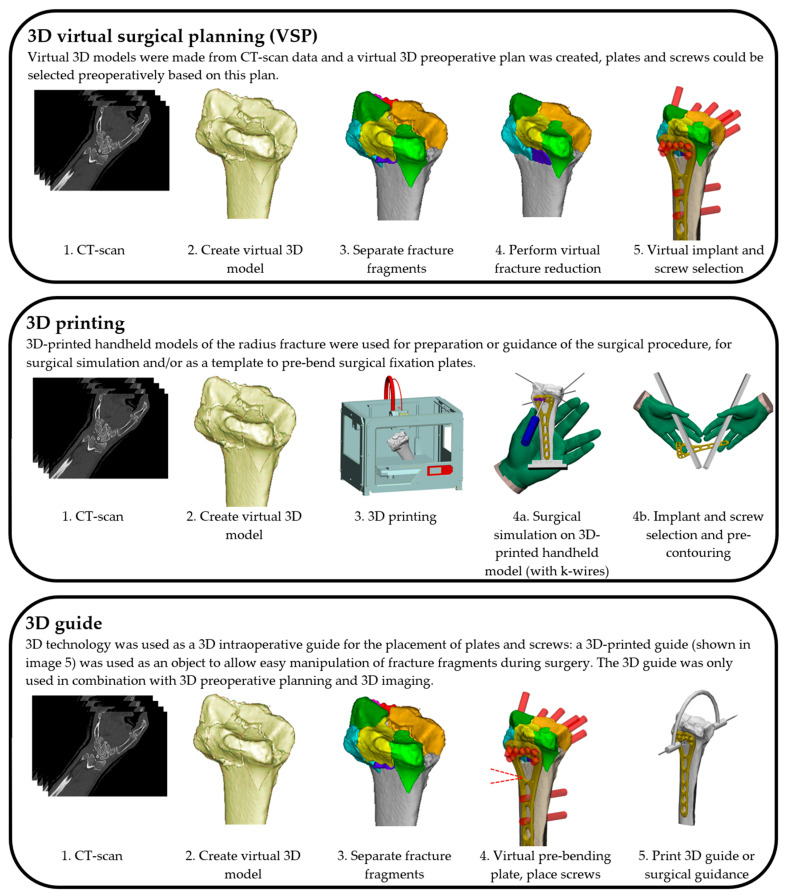
Schematic representation of the 3D applications for distal radius fracture surgery that were found.

Three different concepts of 3D-assisted surgery were identified: (1) 3D virtual surgical planning (‘3D VSP’); (2) 3D-printed handheld models for surgical simulation (‘3D printing’); and (3) the use of 3D intraoperative guides (‘3D guide’). In most studies, only one 3D technology was applied, whereas one study combined two (3D VSP, 3D guide) and one also added a third (3D VSP, 3D printing, 3D guide) technique [14,40]. Figure 2 illustrates the methods of application of each concept in a stepwise manner.

#### 3.3.1. Three-Dimensional Virtual Surgical Planning

**Figure 3:**
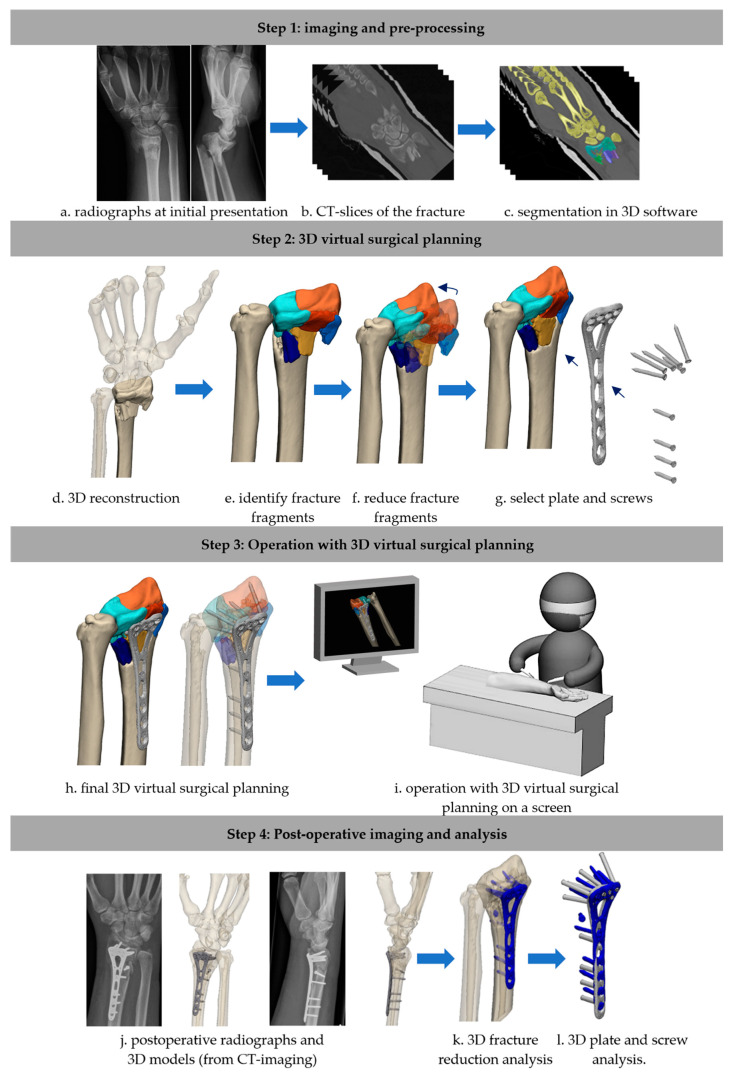
A step-by-step representation of the workflow of 3D virtual surgical planning, and evaluation of the postoperative results. Explanation of colors: Illustration c: yellow, green and blues: masks of the fracture fragments. Illustrations e-i: oranges and blues: fracture fragments, Illustration K and Y: blue: 3D plate and screws model, derived from the post-operative CT scan.

Seven studies (424 patients) reported on the use of 3D VSP (Figure 2) [14,37,38,39,40,41]. The 3D planning software most frequently used was the Zed-Trauma software V1 (Zed-Trauma, LEXI Co., Ltd. Tokyo, Japan), which was developed by the authors for virtual fracture reduction and implant selection. The 3D VSP software was used to visualize fracture displacement, simulate fragment reduction, select and position the plate and screws, and verify the final fracture reduction based on a 3D distal radius template, or mirrored contralateral radius [37,38,39,40,41]. To clarify the 3D VSP process further, Figure 3 has been added, presenting a more elaborate display of the 3D virtual surgical planning workflow, and the 3D postoperative analysis as performed in our center.

#### 3.3.2. Three-Dimensional Printing

Three-dimensionally printed handheld models were used in six studies (315 patients) for surgical simulation [32,33,35] (Figure 2). Chen et al. used the Materialise Mimics software v10.01 (Materialise, Leuven, Belgium) to reconstruct CT images and create 3D models [32,33]. These were transferred as STL (Surface Tessellation Language) files to a 3D printer. Preoperatively, the full-scale 3D-printed handheld model of the fracture was used to manually simulate fracture reduction and predetermine plate and screw sizes and positions. Gui et al. [35] used the same software and printed a 3D life-size model of the fracture to identify the best location for K-wires to reduce hard-to-reach fragments [35]. Kong et al. [20] applied the same method but additionally used a mirrored 3D-printed model of the contralateral radius for pre-bending the surgical plates.

#### 3.3.3. Three-Dimensional Intraoperative Guides

Two studies (63 patients) reported on the use of 3D intraoperative guides [14,40], both in combination with one or more other surgical 3D techniques (Figure 2). Yoshii et al. applied a combination of 3D intraoperative guidance and 3D virtual surgical planning [40]. The virtual planning was then used intraoperatively to create an overlay with real-time fluoroscopy images [14]. Overlapping of the bone and plate contours was used to guide fracture reduction and implant placement [40]. The second study by Xu et al. described the use of 3D handheld printed models in addition to 3D VSP and 3D guides [14]. First, the fracture fragments were reduced in the virtual 3D model. Then, this model was printed and used as a template for screw and plate fitting. An additional 3D-printed K-wire guide was used for 3D imaging-controlled fracture reduction [14].

### 3.4. Effect of 3D-Assisted Surgery on Intraoperative Metrics

Five studies reported on at least one intraoperative metric, of which four used 3D-printed handheld models, and one used 3D virtual surgical planning [20,32,33,34,37]. Results are presented in Table 4.

#### 3.4.1. Operation Time

Operation time was reported in five studies, analyzing 302 patients (Table 4) [20,32,33,34,37]. The weighted means of the studies reporting operation time were 73.2 (63.5–101.8) minutes in the 2D group and 66.9 (51.4–95.3) minutes in the 3D-assisted group. The evidence regarding the effect of 3D printing is strong [27].

#### 3.4.2. Fluoroscopy Frequency

The intraoperative fluoroscopy frequency was assessed in three studies (192 patients) [20,32,33]. 3D printing reduced the fluoroscopy frequency from 5.8 (range 5.6–5.9) in the conventional group to 4.7 (range 4.2–4.9) in the 3D print group (*p* < 0.05 in all three studies) (Table 4). The evidence was moderate for a reduction in fluoroscopy frequency of 1.1 images per operation with 3D assistance [27].

### 3.5. Effect of 3D-Assisted Surgery on Postoperative Outcomes

All twelve studies included in this review reported on postoperative outcomes [14,20,32,33,34,35,36,37,38,39,40,41], of which seven compared a 2D and a 3D group. Data for each group of outcomes is displayed in Table 5.

#### 3.5.1. Patient Follow-Up: PROMs

Two studies (98 patients) analyzed differences between the PROMs in the conventional and 3D groups (Table 5) [20,34]. The weighted mean of the DASH score was 23.9 in the conventional 2D group and 17.8 points in the 3D-assisted group. However, this difference was only reflected in a low-quality study, which showed a 9-point lower DASH in the 3D-assisted group compared to the conventional group (23.6 ± 20.2 vs. 14.9 ± 13.1, respectively), while the comparative high-quality study showed no differences between groups [20,34]. Additionally, no statistically significant differences were found in the PRWE (1 study, 19.6 ± 20.8 vs. 13.8 ± 2.4; *p* = 0.342) or the VAS pain score (2 studies, weighted mean = 1.0 (0.9–1.0) vs. 0.8 (0.8–0.9)), and the evidence, according to the best evidence synthesis, was conflicting [20,27,34].

#### 3.5.2. Patient Follow-Up: Function/Range of Motion

Differences between 2D- and 3D-assisted surgery in wrist extension, flexion, pronation and supination or combined follow-up measurements in the Gartland Werley score were analyzed in four studies (253 patients). No significant differences were found in any of these outcome measures (Table 5) [20,32,33,34]. The weighted mean of the measured differences in function/ROM was within several degrees between the 2D- and 3D-assisted groups. These differences are not clinically relevant, and evidence on function/ROM was moderate [27].

#### 3.5.3. Fracture Reduction

Eleven studies (671 patients) reported on intra-articular fracture reduction (e.g., reduction satisfaction, ulnar deviation, palmar tilt, radial height, ulnar variance and, radial inclination), of which seven compared a 2D and a 3D group (526 patients) (Table 5) [20,32,33,34,36,39,40]. There was no difference in fracture reduction between the 3D-assisted surgery and the conventional group in one study (94 vs. 94% satisfactory rate) [20]. Three studies, including one high-quality study, found small differences in palmar tilt, ulnar variance and radial inclination, which were not clinically relevant (0.3 to 1.4 degrees) [14,38,39]. The weighted mean was calculated for the 2D and 3D groups, but no differences were found in any of the parameters defining fracture reduction. Evidence on fracture reduction satisfaction, according to the best evidence synthesis, was conflicting or moderate [27].

#### 3.5.4. Complication Rate

Two studies (98 patients) compared complication rates between a 2D and a 3D group (Table 5) [20,34]. Among these complications were screw and/or plate removal due to irritation, secondary fracture displacement, wound infection, carpal tunnel syndrome, wound dehiscence, complex regional pain syndrome and tendon injury [20,34]. The weighted mean of the complication rate was 3.6% in the 3D assisted group and 10.7% in the conventional group. However, the level of evidence is conflicting, according to the best evidence synthesis [27].

#### 3.5.5. Accurate Length of Placed Screws

Only one comparative study (49 patients) measured the total rate of successful screw placement (%), which was defined as being not intra-articular and being within the range of 75–100% of the planned screw length, or not penetrating the second bone surface by more than 2mm (Table 5) [37]. Successful screw placement was achieved in 75% of the conventional group and in 86% of the 3D-assisted group. Level of evidence was conflicting, according to the best evidence synthesis [27].

## 4. Discussion

To date, a comprehensive overview and critical appraisal of 3D-assisted technology used in distal radius fracture treatment has been lacking. To the best of our knowledge, the review of Zhu et al. is the only one available, but it only addresses 3D printing [15]. In this systematic review, we aimed to summarize all available studies on 3D technologies and to analyze their effects on intraoperative and postoperative outcome measures. The twelve articles that were included describe three different 3D technologies: (1) 3D virtual surgical planning; (2) 3D printing; and (3) 3D surgical guides. The quality of the included articles, according to the McMaster assessment, was moderate to excellent, and five articles were considered high-quality according to the best evidence synthesis [27]. Additionally, there was substantial heterogeneity in the study set-up and outcome measures in the results of the included studies. Regarding intraoperative metrics, the use of 3D-printed handheld models for surgical planning demonstrated a reduction in operation time and a reduced fluoroscopy frequency. Regarding postoperative outcomes, 3D-assisted, as compared to conventional 2D-assisted distal radial fracture surgery, showed no improvements in PROMs, ROM, or fracture reduction. Although, it likely reduces the complication rate and improves the use of appropriate screw lengths.

The first research question addressed the effect of 3D technology on intraoperative metrics [20,32,33,34,36]. This review showed that the use of 3D handheld models reduces operation time by a mean of 6.3 min [20,32,33,34,37], and the fluoroscopy frequency by a mean of 1.1 images per operation [20,32,33], which is in line with the review performed by Zhu et al. [15]. The use of 3D technologies is expected to enhance the surgical process by enabling more thorough preparation and providing an improved preoperative understanding of the fracture, which could explain these results. This finding was primarily based on data obtained from studies that used 3D-printed handheld models for surgical planning. In summary, there is moderate to strong evidence that the use of 3D-printed handheld models reduces operation time and fluoroscopy frequency. Data on the application of 3D virtual surgical planning and 3D guides are limited.

The second research question focused on the possible benefits of 3D-assisted surgical techniques on postoperative outcomes [14,20,32,33,34,35,36,37,38,39,40,41]. We hypothesized that 3D technologies—virtual, printed, or guided—might optimize surgical planning, reduction strategies and fracture fixation, resulting in an improved clinical outcome. However, our comprehensive review revealed that the evidence supporting these potential benefits is still limited. Various PROMs (DASH, PRWE, VAS pain) were used in the 2D- and 3D-assisted groups, with a limited number of patients per study, making it difficult to compare techniques [20,34]. The weighted mean difference in DASH scores between 2D and 3D groups was 9 points, which is below the minimal clinically important difference (MCID) of 10.8 points [42]. A slight improvement in the PRWE was only mentioned in one study, and the difference in the weighted mean of the VAS score was only 0.2 points [20,34]. The application of 3D printing showed some differences in the range of motion; however, the measured differences were minimal (up to 5 degrees) and therefore clinically not significant [20,32,33,34]. Comparative studies found no clinically significant differences in fracture reduction between 2D and 3D groups [20,32,33,34,36,39,40]. 3D techniques may reduce complication rates, as indicated in one high-quality and one low-quality study [20,34]. No cases of fracture nonunion were reported following the use of 3D-assisted surgery. Regarding screw placement, one high-quality study found improved screw selection in 3D-assisted surgery, which is relevant for reducing the risk of complications due to penetrating screws or secondary fracture dislocation [37]. In summary, evidence is still insufficient to show that 3D techniques improve PROMs, ROM, or fracture reduction. However, evidence from this review mildly suggests that they may help lower complication rates and improve screw placement. Further research is necessary to assess the impact of 3D-assisted surgery on clinical outcomes. Future studies on 3D assessment of initial and residual fracture displacement are needed to make data-driven treatment decisions [43].

This review has some strengths and limitations. One of the strengths is that it is one of the few works providing a comprehensive overview of 3D technologies used in the treatment of acute distal radial fractures. The main limitation of this review is that it relies on a small number of studies that address various 3D technologies, including 3D virtual surgical planning, 3D-printed handheld models, and 3D-printed intraoperative guides. Furthermore, these studies show differences in their methodologies and outcome measures, which prevents the possibility of performing a meta-analysis. We therefore believe that future studies are necessary to assess the benefits of these 3D techniques on both intraoperative and postoperative outcomes.

## 5. Conclusions

In conclusion, the use of 3D technologies in distal radial fracture surgery aims to enhance the surgical process by enabling more detailed planning and providing a better preoperative understanding of the fracture pattern, reduction strategy, plate positioning, and optimal screw lengths. This approach supports the principle of “plan your operation and operate your plan”. While 3D-assisted surgery has been shown to slightly reduce operation time and the frequency of fluoroscopy, evidence for significant improvements in fracture reduction quality and functional outcomes is not provided in current studies. However, it likely helps decrease the complication rate (screw and/or plate removal due to irritation, secondary fracture displacement, wound infection, carpal tunnel syndrome, wound dehiscence, complex regional pain syndrome and tendon injury) and enhances the accuracy of selecting appropriate screw lengths.

## Figures and Tables

**Table 1:** Search strategy.

Database	Search String
**Pubmed**	(3D[tiab] OR three dimension*[tiab] OR 3 dimension*[tiab] OR ‘Printing, Three-Dimensional’ [Mesh] OR ‘Imaging, Three-Dimensional’ [Mesh]) AND (Radius*[tiab] OR ‘Radius’ [Mesh]) AND (fractur*[tiab] OR ‘Fractures, Bone’ [Mesh]) AND ‘2010/01/01’ [PDat]: ‘3000/12/31’ [PDat]
**Embase**	(‘three dimensional imaging’/exp OR ‘three dimensional printing’/exp OR ‘3 d’:ti,ab OR ‘3 dimension*’:ti,ab OR ‘three dimension*’:ti,ab) AND (‘radius’/exp OR radial*:ti,ab) AND (‘fracture’/exp OR fractur*:ti,ab) AND [embase]/lim AND [2010–2024]/py

Full search string for both databases used to identify all applicable articles on the subject of this review. * truncation symbol which allows looking for all variations of the word.

**Table 2:** Study characteristics.

Study	Year	Country	N	Population	Period	3D Technology *	Outcome Measurements **
Chen et al. [32]	2018	China	107	Die punch fractures	2013–2015	3D prints	Primary: patient follow-up, fracture reduction Secondary: operation time, N fluoroscopy
Chen et al. [33]	2019	China	48	AO23C	2014–2015	3D prints	Primary: patient follow-up, fracture reduction Secondary: operation time, N fluoroscopy
Grinčuk et al. [34]	2023	Lithuania	66	AO23C	2021–2022	3D prints	Primary: patient follow-up, fracture reduction, complication rate Secondary: Operation time
Gui et al. [35]	2021	China	41	AO23C2 + C3 with dorsal comminution	2017–2018	3D prints	Primary: patient follow-up, fracture reduction, complication rate Secondary: -
Kong et al. [20]	2020	China	32	AO23B + C	2017–2018	3D prints	Primary: patient follow-up, fracture reduction, complication rate Secondary: operation time, N fluoroscopy
Kohyama et al. [36]	2024	Japan	171	AO23A3,B3 + C	2014–2021	3D VSP	Primary: fracture reduction Secondary: -
Totoki et al. [37]	2018	Japan	49	AO23A2-A3 + C	Not reported	3D VSP	Primary: accuracy of screw placement Secondary: operation time
Xu et al. [14]	2019	China	21	AO23C	2017–2018	3D VSP, 3D prints, 3D guide	Primary: patient follow-up, fracture reduction, complication rate, accuracy of screw placement Secondary: -
Yoshii et al. [38]	2017	Japan	20	AO23A2-A3 + C	Not reported	3D VSP	Primary: fracture reduction, accuracy of screw placement Secondary: -
Yoshii et al. [39]	2018	Japan	60	AO23A2-A3 + B3 + C	Not reported	3D VSP	Primary: fracture reduction Secondary: -
Yoshii et al. [40]	2019	Japan	42	AO23A3 + C2-C3	Not reported	3D VSP, 3D guide	Primary: fracture reduction, accuracy of screw placement Secondary: -
Yoshii et al. [41]	2021	Japan	63	AO23A3 + C2-C3	2015-2019	3D VSP	Primary: patient follow-up, fracture reduction Secondary: -

* 3D technologies are described in Figure 2. ** Outcome measures: Radiological measurements of fracture reduction consist of intra-articular displacement measured on a CT scan and radial inclination/ulnar deviation (degrees), palmar/dorsal/volar tilt/angulation (degrees), radial height (mm), ulnar variance (mm) measured on radiographs. Patient follow-up consists of function/range of motion: Gartland–Werley wrist score, wrist extension, flexion, pronation, supination and ulnar and radial deviation, and patient-reported outcome measures: DASH, PRWE and VAS.

**Table 3:** McMaster’s quality assessment.

	Chen 2018 [32]	Chen 2019 [32]	Grinčuk 2023 [34]	Gui 2021 [35]	Kong 2020 [20]	Kohyama 2024 [36]	Totoki 2018 [37]	Xu 2020 [14]	Yoshii 2017 [38]	Yoshii 2019 [40]	Yoshii 2018 [39]	Yoshii 2021 [41]
1. Study purpose												
Was the study question clearly stated?	1	1	0	1	0	1	1	1	1	0	1	1
2. Literature review												
Was relevant background literature reviewed?	0	1	0	0	1	0	1	0	0	1	1	1
3. Study design *	RCT	RCT	RCT	CSD	RCT	CCD	COH.	COH.	COH.	RCT	RCT	CSD
4. Sample												
Was the sample described in detail?	1	1	1	1	1	0	1	1	1	1	1	1
Was the sample justified?	0	0	0	0	1	0	1	0	0	0	0	0
Were the groups randomized?	1	1	1	NA	1	NA	NA	NA	NA	1	0	NA
Was randomizing appropriately done?	0	0	1	NA	1	NA	NA	NA	NA	0	0	NA
5. Outcomes												
Were the outcome measures reliable?	0	0	1	1	1	1	1	1	1	0	0	1
Were the outcome measures valid?	0	0	1	1	1	1	0	1	1	0	1	0
6. Intervention												
Intervention was described in detail?	1	0	1	1	1	1	1	0	1	1	1	1
Contamination was avoided?	1	1	0	NA	1	0	1	NA	NA	1	0	NA
Cointervention was avoided?	0	0	1	NA	1	1	1	NA	NA	1	0	NA
7. Results												
Results were reported in terms of statistical significance?	1	1	1	1	1	1	1	1	1	1	1	1
Were the analysis method/s appropriate?	1	1	0	1	1	1	1	1	1	1	0	1
Clinical importance was reported?	1	1	0	1	1	1	1	1	0	0	1	0
Drop-outs were reported?	1	1	0	1	0	0	1	0	1	0	0	0
8. Conclusion												
Conclusions were appropriate given study methods and results?	0	0	1	1	1	1	1	1	1	1	1	1
Total n points	9	9	9	10	14	9	13	8	9	9	8	8
Maximum points possible	16	16	06	12	16	14	14	12	12	16	16	12
Quality score (%)	56	56	56	83	88	64	93	67	75	56	50	67
Study quality interpretation **	M	M	M	G	G	M	E	G	G	M	M	M
High Quality study? ***				✓	✓		✓	✓	✓			

Questions are answered with ‘yes’ (1 point), ‘no’ (0 points) or ‘NA’ if the question was not applicable to the study design. *Abbreviations of study type according to the McMaster University Occupational Therapy Evidence-Based Practice Research Group guideline: RCT: randomized controlled trial, CSD: cross-sectional design, CCD: case–control design, COH: cohort study. ** Abbreviations used to indicate study quality interpretation: ‘M’ stands for ‘Moderate’, ‘G’ stands for ‘Good’ and ‘E’ stands for ‘Excellent’ *** High-quality studies according to the best evidence synthesis are indicated with a ‘✓’ in the last row of this table.

**Table 4:** Study outcomes: Intraoperative metrics (primary outcomes).

**Operation Time**							
Measure	Study	3D Technology	Groups		Outcome ± SD		
			2D (N)	3D (N)	2D	3D	Sig. (*p*)
Operation Time (min)	Chen 2018 [32]	3D printing	55	52	65.7 ± 6.0	56.6 ± 4.7	*p* < 0.001 *
	Chen 2019 [33]	3D printing	25	23	75.4 ± 6.0	66.5 ± 5.3	*p* < 0.001 *
	Grinčuk 2023 [34]	3D printing	33	33	72.1 ± 17.5	65.1 ± 15.2	*p* = 0.072
	Kong 2020 ✓ [20]	3D printing	16	16	63.5 ± 5.9	51.4 ± 6.8	*p* < 0.001 *
	Totoki 2018 ✓ [37]	3D VSP	19	28	101.8 ± NA	95.3 ± NA	*p* > 0.05
	Sum N patients/Weighted mean (range)		148	154	73.2 (63.5–101.8)	66.9 (51.4–95.3)	
**Fluoroscopy Frequency**							
Measure	Study	3D Technology	Groups		Outcome ± SD		
			2D (N)	3D (N)	2D	3D	Sig. (*p*)
Fluoroscopy frequency (N)	Chen 2018 [32]	3D printing	55	52	5.9 ± 1.6	4.9 ± 1.4	*p* < 0.001 *
	Chen 2019 [33]	3D printing	25	23	5.6 ± 1.6	4.4 ± 1.4	*p* = 0.011 *
	Kong 2020 ✓ [20]	3D printing	16	16	5.6 ± 1.1	4.2 ± 1.3	*p* = 0.002 *
	Sum N patients/Weighted mean (range)		96	96	5.8 (5.6–5.9)	4.7 (4.2–4.9)	

NA = Not Addressed; * and bold = Statistically significant outcome; ✓ = indicates a high-quality study; Studies displayed in grey are not considered in the weighted mean calculation due to a difference in display of the outcome measure or due to comparison to another group than the standard conventional 2D group.

**Table 5:** Study outcomes: Postoperative measures (secondary outcomes).

**Patient Follow-Up: PROMs**								
Measure	Study		3D Technology	Groups		Outcome ± SD		
				2D (N)	3D (N)	2D	3D	Sig. (*p*)
DASH ± SD	Kong 2020 ✓ [20]		3D printing	16	16	24.5 ± 7.0	23.8 ± 8.1	*p* = 0.91
	Grinčuk 2023 [34]		3D printing	33	33	23.6 ± 20.2	14.9 ± 13.1	*p* = 0.094
Sum N patients/Weighted mean (range)				49	49	23.9 (23.6–24.5)	17.8 (14.9–23.8)	
PRWE ± SD	Grinčuk 2023 [34]		3D printing	33	33	19.6 ± 20.8	13.8 ± 2.4	*p* = 0.342
Sum N patients/Weighted mean (range)				33	33	19.6	13.8	
VAS ± SD	Kong 2020 ✓ [20]		3D printing	16	16	0.9 ± 0.3	0.9 ± 0.2	*p* = 0.80
	Grinčuk 2023 [34]		3D printing	33	33	1.0 ± 1.3	0.8 ± 0.8	*p* = 0.554
Sum N patients/Weighted mean (range)				49	49	1.0 (0.9–1.0)	0.8 (0.8–0.9)	
**Patient Follow-Up: Function/ROM: (Difference Between Uninjured and Fractured Side for the 2D and 3D Group)**								
Measure	Study		3D Technology	Groups		Outcome ± SD		
				2D (N)	3D (N)	2D	3D	Sig. (*p*)
Gartland–Werley score ± SD (pts)	Chen 2018 [32]		3D printing	55	52	86.0 ± 15.2	86.4 ± 14.1	*p* = 0.878
	Chen 2019 [33]		3D printing	25	23	74.8 ± 16.6	75.7 ± 15.5	*p* = 0.211
	Sum N patients/Weighted mean (range)			80	75	82.5 (74.8–86.0)	83.1 (75.7–86.4)	
Extension ± SD (Δdeg.)	Chen 2018 [32]		3D printing	55	52	4.6 ± 3.7	4.6 ± 3.8	*p* = 0.993
	Chen 2019 [33]		3D printing	25	23	3.8 ± 3.1	4.1 ± 3.5	*p* = 0.765
Sum N patients/Weighted mean (range)				80	75	4.3 (3.8–4.6)	4.4 (4.1–4.6)	
Flexion ± SD (Δdeg.)	Chen 2018 [32]		3D printing	55	52	4 ± 3.7	3.6 ± 3.2	*p* = 0.606
	Chen 2019 [33]		3D printing	25	23	3.6 ± 2.7	3.1 ± 2.7	*p* = 0.511
	Grinčuk 2023 [34]		3D printing	33	33	12.1 ± 10.1	7.5 ± 5.9	*p* = 0.06
	Sum N patients/Weighted mean (range)			113	108	6.6 (3.6–12.1)	4.7 (3.1–7.5)	
Pronation ± SD (Δdeg.)	Chen 2018 [32]		3D printing	55	52	5.5 ± 3.9	5.5 ± 3.4	*p* = 0.906
	Chen 2019 [33]		3D printing	25	23	4.5 ± 3.7	5.1 ± 3.2	*p* = 0.548
	Grinčuk 2023 [34]		3D printing	33	33	1.7 ± 5.1	1.0 ± 2.9	*p* = 0.926
Sum N patients/Weighted mean (range)				113	108	4.1 (1.7–5.6)	4.0 (1–5.5)	
Supination (Δdeg.)	Chen 2018 [32]		3D printing	55	52	5.6 ± 4.3	5.3 ± 4.7	*p* = 0.671
	Chen 2019 [33]		3D printing	25	23	4.9 ± 3.3	4.4 ± 3.3	*p* = 0.613
	Grinčuk 2023 [34]		3D printing	33	33	2.0 ± 5.3	1.3 ± 4.5	*p* = 0.692
Sum N patients/Weighted mean (range)				113	108	4.4 (2.0–5.6)	3.9 (1.3–5.3)	
Radial deviation ± SD (deg.)	Kong 2020 ✓ [20]		3D printing	16	16	23.2 ± 4.9	24.8 ± 5.1	*p* = 0.38
	Sum N patients/Weighted mean (range)			16	16	23.2	24.8	
Ulnar deviation ± SD (deg.)	Kong 2020 ✓ [20]		3D printing	16	16	19.8 ± 5.8	22.0 ± 6.9	*p* = 0.35
	Grinčuk 2023 [34]		3D printing	33	33	13.8 ± 10.4	9.5 ± 7.1	*p* = 0.12
Sum N patients/Weighted mean (range)				49	49	15.7 (13.6–19.8)	13.6 (9.5–22.0)	
**Fracture Reduction (Difference Between Postoperative Fractures Side and Contralateral, or Direct** **Measurement of Reduced Fracture)**								
Measure	Study		3D Technology	Groups		Outcome ± SD		
				2D (N)	3D (N)	2D	3D	Sig. (*p*)
Satisfying Intra-articular fracture reduction rate (%)	Kong 2020 ✓ [20]		3D printing	16	16	94	94	*p* > 0.05
	Sum N patients/Weighted mean (range)			16	16	94	94	
Ulnar deviation (deg.)	Chen 2018 [32]		3D printing	55	52	20.7 ± 1.8	20.6 ± 1.8	*p* = 0.868
	Chen 2019 [33]		3D printing	25	23	20.4 ± 1.5	20.9 ± 1.7	*p* = 0.309
Sum N patients/Weighted mean (range)				80	75	20.6 (20.4–20.8)	20.7 (20.6–20.9)	
Ulnar deviation (Δdeg.)	Grinčuk 2023 [34]		3D printing	33	33	1.2 ± 2.3	0.3 ± 1.8	*p* = 0.254
	Kohyama 2024 [36]		3D VSP	108	63	1.4 ± 1.4	1.3 ± 1.0	*p* = 0.71
	Yoshii 2019 [40]		3D VSP, 3D guide	21	21	1.6 ± 1.6	1.7 ± 1.1	NA
Sum N patients/Weighted mean (range)				162	117	1.4 (1.2–1.6)	1.1 (0.3–1.7)	
Palmar tilt (deg.)	Chen 2018 [32]		3D printing	55	52	12.7 ± 1.6	12.4 ± 1.4	*p* = 0.467
	Chen 2019 [33]		3D printing	25	23	12.7 ± 1.9	12.2 ± 1.5	*p* = 0.359
Sum N patients/Weighted mean (range)				80	75	12.7 (12.7–12.7)	12.3 (12.2–12.4)	
Palmar tilt (Δdeg.)	Yoshii 2019 [40]		3D VSP, 3D guide	21	21	2.5 ± 2.3	2.2 ± 1.7	NA
	Yoshii 2018 [39]		3D VSP	30	30	4.0 ± 3.1	2.5 ± 1.8	*p* = 0.03 *
	Grinčuk 2023 [34]		3D printing	33	33	4.3 ± 8.7	3.7 ± 4.9	*p* = 0.158
Sum N patients/Weighted mean (range)				84	84	3.7 (2.5–4.3)	2.9 (2.2–3.7)	
Radial height (mm.)	Chen 2018 [32]		3D printing	55	52	12.7 ± 1.6	12.4 ± 1.9	*p* = 0.410
	Chen 2019 [33]		3D printing	25	23	12.6 ± 1.8	12.6 ± 1.9	*p* = 0.987
	Grinčuk 2023 [34]		3D printing	33	33	11.8 ± 2.5	10.8 ± 2.7	*p* = 0.161
Sum N patients/Weighted mean (range)				113	108	12.4 (11.7–12.7)	12.0 (10.8–12.6)	
Ulnar variance (mm)	Kohyama 2024 [36]		3D VSP	108	63	0.8 ± 0.8	0.4 ± 0.6	*p* = 0.02 *
	Yoshii 2018 [39]		3D VSP	30	30	1.10 ± 0.94	0.92 ± 0.76	*p* > 0.05
Sum N patients/Weighted mean (range)				138	93	0.9 (0.8–1.1)	0.6 (0.4–0.9)	
Radial inclination (Δdeg.)	Kohyama 2024 [36]		3D VSP	108	63	1.3 ± 1.0	1.2 ± 1.2	*p* = 0.69
	Yoshii 2018 [39]		3D VSP	30	30	2.00 ± 1.58	3.40 ± 3.00	*p* = 0.03 *
Sum N patients/Weighted mean (range)				138	93	1.45 (1.3–2.00)	1.91 (1.2–3.40)	
Radial inclination (deg.)	Grinčuk 2023 [34]		3D printing	33	33	22.56 ± 7.9	24.9 ± 6.2	*p* = 0.17
Sum N patients/Weighted mean (range)				33	33	22.5	24.9	
**Complication Rate**								
Measure	Study		3D Technology	Groups		Outcome ± SD		
				2D (N)	3D (N)	2D	3D	Sig. (*p*)
Complication rate (%)	Kong 2020 ✓ [20]		3D printing	16	16	12	7	NA
	Grinčuk 2023 [34]		3D printing	33	33	10	2	*p* = 0.02 *
Sum N patients/Weighted mean (range)				49	49	10.7 (10–12)	3.6 (2–7)	
**Accuracy Screw Placement**								
Measure	Study		3D Technology	Groups		Outcome ± SD		
				2D (N)	3D (N)	2D	3D	Sig. (*p*)
Accurate length of placed screws (%)		Totoki 2018 ✓ [37]	3D VSP	21	28	75	86	*p* < 0.05 *
		Sum N patients/Weighted mean (range)		21	28	75	86	-

NA = Not Addressed; * and bold = Statistically significant outcome; ✓ = indicates a high-quality study; Studies displayed in grey are not considered in the weighted mean calculation due to a difference in display of the outcome measure or due to comparison to another group than the standard conventional 2D group.

## Data Availability

The authors declare that the data supporting the findings of this study are available within the paper and upon request from the authors.

## List of Abbreviations

2D	Two-dimensional
3D	Three-dimensional
CCD	Case–control design
COH	Cohort study
CSD	Cross-sectional design
CT	Computed tomography
DASH	Disability of the Arm, Shoulder, and Hand
deg.	Degrees
Δdeg.	Difference in degrees
mm	Millimeters
NA	Not addressed/not applicable
PRISMA	Preferred Reporting Items for Systematic Reviews
PROMs	Patient-reported outcome measures
PROSPERO	International prospective register of systematic reviews
PRWE	Patient-Rated Wrist Evaluation
RCT	Randomized controlled trial
ROM	Range of motion
SD	Standard deviation
VAS	Visual analogue scale
VSP	Virtual surgical planning
